# Flight Synchrony among the Major Moth Pests of Cranberries in the Upper Midwest, USA

**DOI:** 10.3390/insects8010026

**Published:** 2017-02-26

**Authors:** Shawn A. Steffan, Merritt E. Singleton, Jayne Sojka, Elissa M. Chasen, Annie E. Deutsch, Juan E. Zalapa, Christelle Guédot

**Affiliations:** 1 United States Department of Agriculture, Agricultural Research Service, 1575 Linden Dr., Madison, WI 53706, USA; Elissa.Chasen@ars.usda.gov (E.M.C.); Juan.Zalapa@ars.usda.gov (J.E.Z.); 2 Department of Entomology, University of Wisconsin, 1630 Linden Drive, Madison, WI 53706, USA; merrittsingleton@gmail.com (M.E.S.); guedot@wisc.edu (C.G.); 3 Lady Bug IPM, LLC, 10107 State Hwy 54, Pittsville, WI 54466, USA; ladybug_ipm@hotmail.com; 4 University of Wisconsin-Extension, Door County, 421 Nebraska St., Sturgeon Bay, WI 54235, USA; adeutsch@co.door.wi.us

**Keywords:** *Acrobasis vaccinii*, blackheaded fireworm, cranberry fruitworm, degree-day, IPM, *Rhopobota naevana*, *Sparganothis sulfureana*

## Abstract

The cranberry fruitworm (*Acrobasis vaccinii* Riley), sparganothis fruitworm (*Sparganothis sulfureana* Clemens), and blackheaded fireworm (*Rhopobota naevana* Hübner) are historically significant pests of cranberries (*Vaccinium macrocarpon* Aiton) in the Upper Midwest (Wisconsin), USA. Their respective natural histories are well documented but correlations between developmental benchmarks (e.g., larval eclosion) and degree-day accruals are not yet known. Treatment timings are critical to the optimization of any given control tactic, and degree-day accrual facilitates optimization by quantifying the developmental status of pest populations. When key developmental benchmarks in the pest life cycle are linked to degree-days, real-time weather data can be used to predict precise treatment timings. Here, we provide the degree-day accumulations associated with discrete biological events (i.e., initiation of flight and peak flight) for the three most consistent moth pests of cranberries in Wisconsin. Moths were trapped each spring and summer from 2003 to 2011. To characterize flight dynamics and average timing of flight initiation, pheromone-baited trap-catch data were tallied for all three pest species within each of seven growing seasons. These flight dynamics were then associated with the corresponding degree-day accumulations generated using the cranberry plant’s developmental thresholds. Finally, models were fit to the data in order to determine the peak flight of each species. The initiation of the spring flight among all three moth species was highly synchronous, aiding in the timing of control tactics; however, there were substantial differences in the timing of peak flight among the moth species. Characterization of the relationship between temperature and pest development allows pest management professionals to target specific life stages, improving the efficacy of any given pest control tactic.

## 1. Introduction

One of the central tenets of any integrated pest management (IPM) program is that pest management decisions should be based not only on knowledge of pest identity and abundance, but also on accurate estimates of pest ontogeny (development) and stage-specific damage potential [1,2,3,4]. Generating information on pest ontogeny and damage potential is facilitated by the use of degree-day models. Such models refine predictions of arthropod development using ambient temperatures [5,6,7]. Because insects are poikilothermic, daily temperature data can be used to predict development via the calculation of heat units, or degree-days (DD). To date, phenological models using DDs have been determined for over five hundred insect pests, across five insect families and several landscape systems [8]. DD accrual is typically initiated after a discrete biological event, referred to as a ‘biofix,’ or a calendar date. DD accumulations can then be linked to specific developmental benchmarks, such as the start of oviposition, larval eclosion, or adult emergence, which create useful, predictive models to better time pest management tactics [9,10,11].

In the cranberry system, there have been relatively few studies producing reliable, predictive models of pest development [12,13], although recent work has established the temperature-mediated development thresholds for two key cranberry insect pests [10,14,15]. The American cranberry, *Vaccinium macrocarpon* Aiton, is a high-value crop, and is one of the very few plants native to North America that is grown commercially [16]. Cranberries have been cultivated in the US since the early 1800s [17] and are currently grown almost exclusively in the US and Canada [16]. Cranberry growers in Wisconsin commonly report that insects are their most significant pest group [18]. Given that Wisconsin represents more cranberry production than all other US states combined [19], significant crop losses due to insect feeding can affect global cranberry supplies.

The insect pests of Wisconsin marshes are diverse, but have historically been dominated by three moth species: (1) cranberry fruitworm, *Acrobasis vaccinii* Riley (Lepidoptera: Pyralidae); (2) sparganothis fruitworm, *Sparganothis sulfureana* Clemens (Lepidoptera: Tortricidae); (3) blackheaded fireworm, *Rhopobota naevana* Hübner (Lepidoptera: Tortricidae). Crop loss attributable to *A. vaccinii* can be as high as 50% in untreated beds [20], and Wisconsin growers have consistently ranked this insect as their top pest [18]. *A. vaccinii* has a single generation in the Upper Midwest, with adults beginning to emerge during bloom (mid/late June) and continuing through July. Control of this pest at any stage is challenging, and it is made particularly troublesome because larvae target fruit and remain within berries while feeding. Inside the berry, larvae are protected from predation and spray treatments, and thus require precisely timed insecticide applications to suppress populations before they enter the fruit. Co-occurrence of peak flight and the cranberry bloom presents yet another obstacle to the management of this pest. The cranberry bloom attracts many pollinators, and these pollinators are key to fruit set, so any insecticide treatment applied during bloom can have consequences for both pollinators and fruit set. Finally, overwintering prepupae and/or pupae of *A. vaccinii* appear to be tolerant of spring flooding as practiced in Wisconsin [21], and biological control does not confer adequate pest suppression in conventional spray programs, given the low parasitism rates commonly observed [22]. Optimization of treatment timing, therefore, is critical for this pest species.

Similarly, *S. sulfureana* populations often require a well-timed treatment in the spring to intercept larvae before they can create protective galleries in which to feed. In Wisconsin, overwintered *S. sulfureana* neonates emerge in May and begin feeding on the new succulent growth. There is a second generation each season [23], and these larvae feed on developing berries, as well as the foliage. In both generations, *S. sulfureana* larvae use silk to web together leaves, stems, and berries, forming a tight, protective gallery [16]. This underscores the need for proper timing of insecticide applications to target this species during narrow windows of opportunity. Like *A. vaccinii*, *S. sulfureana* mortality is not consistently attributable to flooding as it is practiced in Wisconsin, presumably due to the hydrophobic, semi-permeable refuge they build out of silk and plant material [24]. While significant levels of biological control of *S. sulfureana* have been demonstrated in growing regions outside of Wisconsin [25], it is not clear how management tactics impact specific natural enemies.

*R. naevana* also has two generations per season in Wisconsin [26]. It overwinters as an egg, and first-generation larvae begin feeding on foliage late May through early June. Like *S. sulfureana*, *R. naevana* webs together cranberry uprights to create a refuge in which to feed. First-generation adults fly from June to July. Second-generation larvae emerge between mid-June through August, feeding primarily on foliage but also fruit, to some extent. Biological control has not been shown to provide significant control of *R. naevana* [22] but flooding can be effective [27].

To refine pest management strategies, it is important to have a means of predicting the start of the mating flight, as well as the peak of this flight. Peak flight is not only the midway point of the flight, but it serves to approximate the relative size of a population. Our objectives focused on gathering trapping data on all three moth species over many years, allowing us to model trap-catch data as a function of DD accrual. With predictive, temperature-based models, it is then possible to provide cranberry growers with the means to create their own real-time pest development information and allow IPM professionals to better assess the developmental status of multiple pest populations within a cranberry bed, marsh, or region. Specifically, we have determined the DD accumulation at which flight initiation and peak flight occur for *A. vaccinii, S. sulfureana*, and *R. naevana*.

## 2. Materials and Methods

### 2.1. Trapping Moths

Populations of *Acrobasis vaccinii*, *Sparganothis sulfureana*, and *Rhopobota naevana* were monitored at 85 commercial cranberry marshes in the Upper Midwest, distributed across the three major cranberry-growing regions of Wisconsin (i.e., Wood, Monroe, Jackson, Juneau, and Adams Counties). The southernmost area of this growing region included the towns of Tomah and Warrens; the central area of the growing region included the towns of Cranmoor, City Point, Nekoosa, and Babcock; the eastern area represented marshes east of the Wisconsin River, including Adams County. Trapping data were compiled during the growing seasons of 2003, 2005, 2007, 2008, 2009, 2010 and 2011.

Moth populations were assessed using “P-2” traps (Great Lakes IPM, Vestaburg, MI, USA) and commercially available species-specific pheromone-baited lures (Scentry Biologicals, Inc. (Billings, MT, USA), Great Lakes IPM (Vestaburg, MI, USA), Agbio, Inc. (Westminster, CO, USA)). At each site, traps were staked into cranberry beds directly above the cranberry canopy, with approximately one trap per four hectares. Traps were deployed in May, collected every week, and adults were tallied over the course of approximately ten weeks. Pheromone lures were changed biweekly. Mean per-trap counts were derived for each site every week.

### 2.2. Degree-Day Calculations

Site-specific DDs were calculated using daily minimum and maximum temperatures for one location representing each region: Volk (Southern), Wisconsin Rapids (Central), and Hancock (Eastern). Weather data for the Southern and Central regions were reported from local weather stations (accessed via http://www.wunderground.com) and for the Eastern region from the Hancock Agricultural Research station (http://www.soils.wisc.edu/uwex_agwx/awon/daily_clim). For any missing weather data from the two previous sources, daily data was used from volunteer weather observers who transmit their data to the National Weather Forecast Service office in Sullivan, WI (UW Extension 2014b). DD calculations were determined using an online DD calculator (UC-IPM Online 2014) using the single sine calculation method and a horizontal cutoff. DD accrual began 1 March of each year (this start date is used because any accrual prior to 1 March would be minimal in the Upper Midwest, but would allow for the inclusion of occasional warm springs in March/April). To standardize the DD accrual for each insect species, a single pair of temperature thresholds were used, and we chose to use the lower and upper thresholds of the cranberry plant. These thresholds were 5 °C (41 °F) and 29 °C (85 °F) [28].

### 2.3. Modeling of Flight Dynamics

Flight dynamics were analyzed as moths per time-step (i.e., week), and these dynamics were modeled using parabolic functions (second-order polynomial functions; *y* = *ax*^2^ + *bx* + *c*) (SigmaPlot 12.3) because these functions closely approximate the bell-shaped curve of the moth flights. Parabolic models were fit to the trap-catch data of any given flight. Peak flight was determined by solving for the *x*-value associated with the peak of the parabola. This was determined by setting the first-derivative of the parabolic equation equal to zero (*y’* = 0 = 2*ax* + *b*) and solving for *x*. This *x*-value corresponds to the point on the curve at which the tangent would have a slope = 0, which is the maximum value of *y* (peak) for the parabola. Although *S. sulfureana* and *R. naevana* are bivoltine, analyses were confined to the first-generation flights for those two species, which is when pest management efforts tend to be focused.

## 3. Results and Discussion

### 3.1. Flight Initiation, by Species

**Figure 1:**
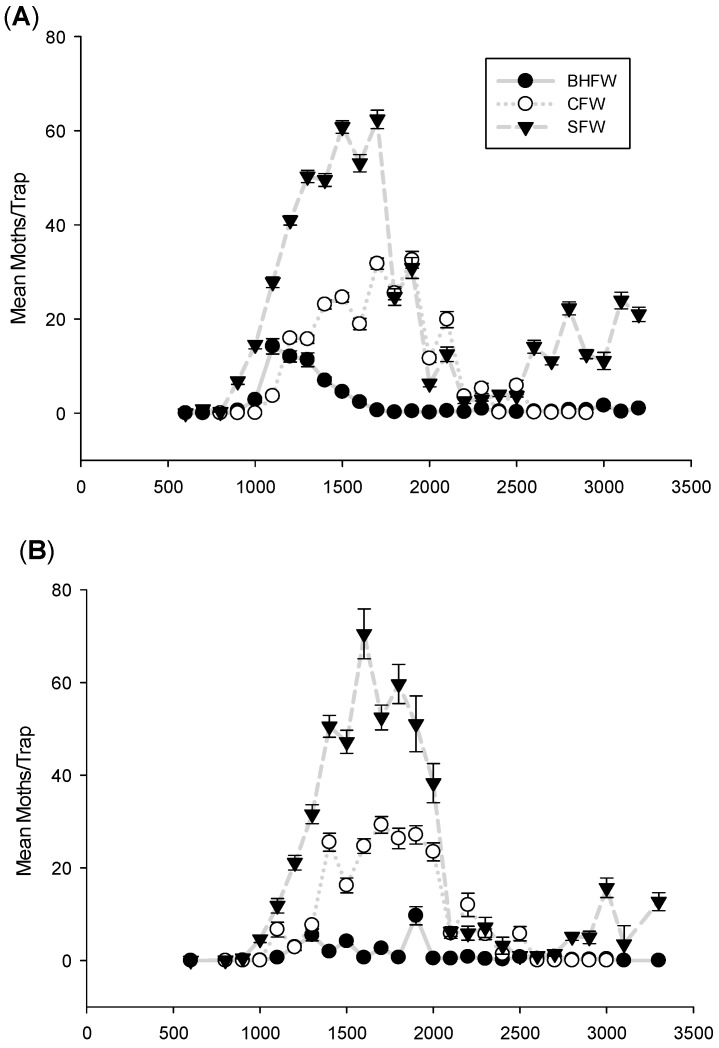

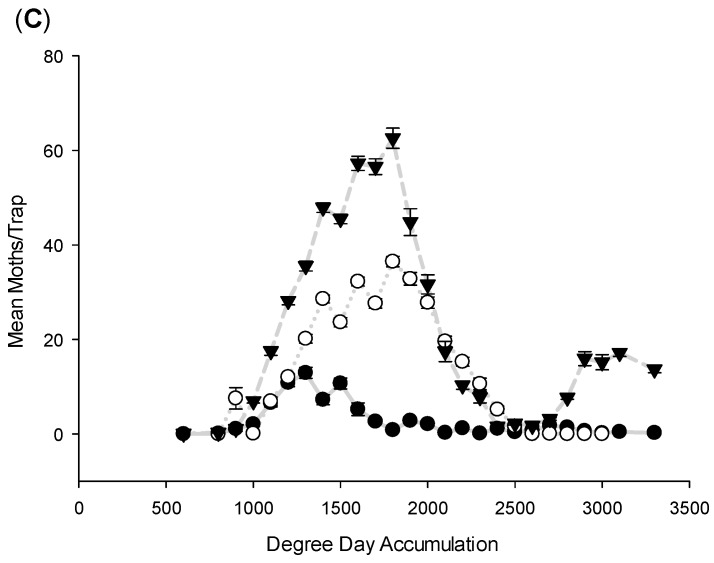
Flights of cranberry fruitworm (CFW), sparganothis fruitworm (SFW), and blackheaded fireworm (BHFW) in the (**A**) southern; (**B**) central; and (**C**) eastern growing regions of Wisconsin.

**Figure 2:**
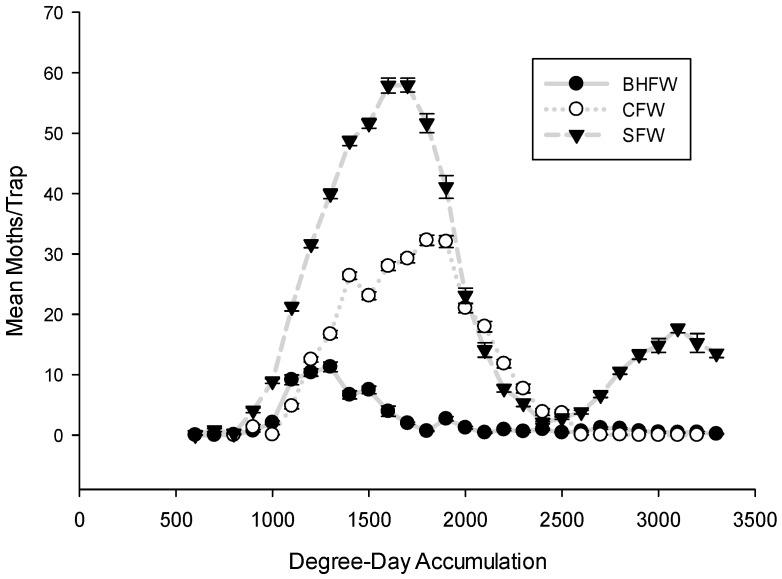
Flights of cranberry fruitworm (CFW), sparganothis fruitworm (SFW), and blackheaded fireworm (BHFW) averaged across all three major growing regions of Wisconsin.

The spring flights *S. sulfureana*, *R. naevana*, and *A. vaccinii* were initiated within a narrow range of DD accumulations in each of Wisconsin’s major growing regions (Figure 1A–C), as well as among regions (Figure 2). The first moth species to begin its spring flight was *S. sulfureana*, appearing in traps typically by 846 DD. The flight of *R. naevana* began soon thereafter at 934 DD, followed by *A. vaccinii*, flying at 997 DD. Thus, these three moth species can be expected to initiate their spring flights within 160 DD of one another, which would correspond to approximately one week, given typical June temperatures in Wisconsin (20–25 DDs can be expected per day). This predictable synchrony in the initiation of flight among these three moth species provides key information for IPM practitioners, particularly when weather or other logistics may preclude adequate scouting.

### 3.2. Peak Flight, by Species

**Figure 3:**
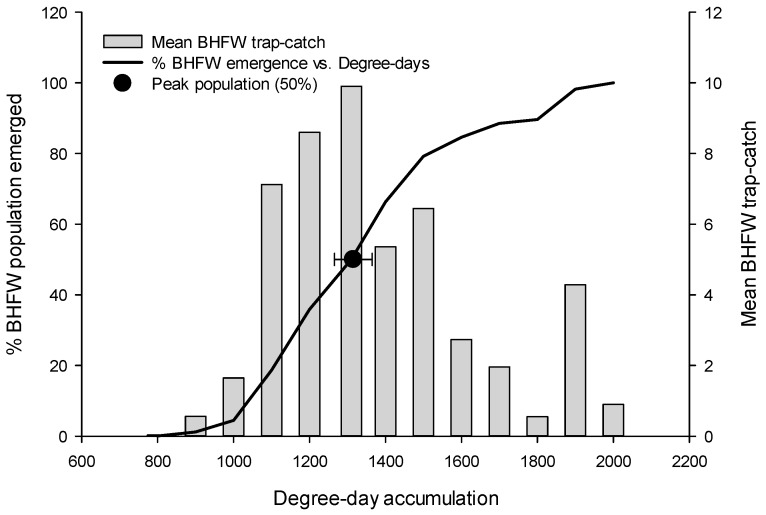
Blackheaded fireworm (BHFW) flight (% emergence, trap-catch, and peak flight) plotted as a function of degree-day accumulation. The peak flight interval (the halfway point, indicated by the black circle, ±95% CI) represents the range of degree-days within which peak flight is most likely to occur.

Despite our observations that all three moth flights were relatively synchronous in their respective commencements, and that the flights overlapped to a high degree (Figure 2), each species exhibited markedly different peaks (Table 1). *R. naevana* was generally the first species to reach its peak. On average, this flight peaked around 1360 DDs (95% CI: 1331–1384 DDs), meaning that 95% of the time, the *R. naevana* peak can be expected to occur between 1331 and 1384 DDs (Table 1 and Figure 3).

**Figure 4:**
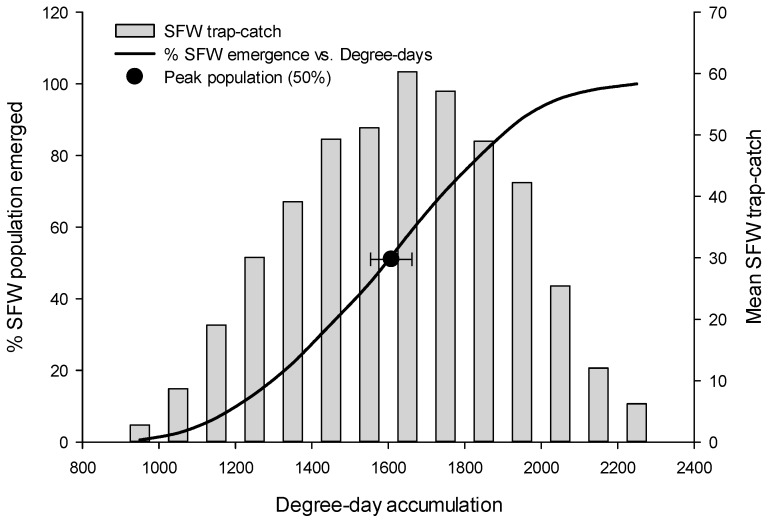
Sparganothis fruitworm (SFW) flight (% emergence, trap-catch, and peak flight) plotted as a function of degree-day accumulation. The peak flight interval (the halfway point, indicated by the black circle, ±95% CI) represents the range of degree-days within which peak flight is likely to occur.

The next moth flight to reach its peak was *S. sulfureana*, occurring at approximately 1610 DDs (95% CI: 1549–1669 DDs) (Table 1 and Figure 4), which was on average 250 DD later than *R. naevana* (~10–14 days later in typical June or July temperatures in central Wisconsin). Again, there will always be year-to-year and site-to-site variability, but the majority of the time, the *S. sulfureana* peak flight can be expected to occur between 1549 and 1669 DDs (Figure 4).

**Figure 5:**
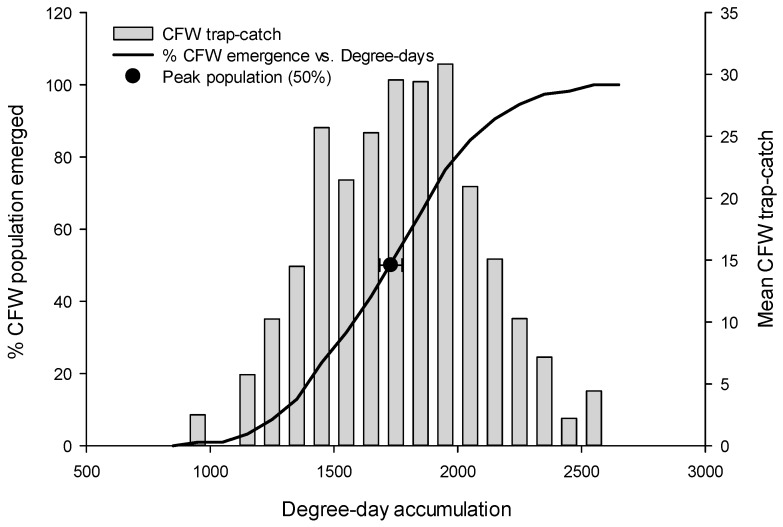
Cranberry fruitworm (CFW) flight (% emergence, trap-catch, and peak flight) plotted as a function of degree-day accumulation. The peak flight interval (the halfway point, indicated by the black circle, ±95% CI) represents the range of degree-days within which peak flight is likely to occur.

The *A. vaccinii* flight was last to peak, and this tended to occur around 1760 DD (95% CI: 1717–1805) (Table 1 and Figure 5), approximately 400 DDs after *R. naevana*, and 150 DDs after *S. sulfureana*. This long delay in the *A. vaccinii* peak flight could be as much as three weeks, complicating efforts to effectively target the adult (the egg-laying stage) of all three species with a single spray. As growers and pest control professionals plan their treatment options, they can expect that 95% of the time, the *A. vaccinii* peak will occur between 1717 and 1805 DDs (Figure 5). Interestingly, despite rather large differences in DD accumulations among the three moth species, this pattern was consistent among the three southern, central, and eastern growing regions of Wisconsin (Figures S7–S9). All model parameter values and figures, by year and region, are archived within the Supplementary Materials (Tables S1–S3).

## 4. Conclusions

Understanding the relationship between insect pest development and ambient temperature improves the timing of scouting efforts and pest management tactics. Historically, there have been three moth species (*A. vaccinii*, *S. sulfureana*, *R. naevana*) that are particularly problematic for cranberry growers in the Upper Midwest of the USA, and while much is known of their respective natural histories, there have been few studies of the associations between degree-day (DD) accumulations and key biological events (e.g., larval eclosion, initiation of flight, and peak flight). Here, we use the developmental thresholds of the cranberry plant as a standardized proxy for the three insect species. We tallied pheromone-baited trap-catch data across seven years and 85 sites throughout central Wisconsin to determine the initiation of flight. We then modeled the flight dynamics, demonstrating that moth trap-catch over time represented a predictable, parabolic function. Finally, the historical peak flights of these three moth species were calculated for each of the three major growing regions in Wisconsin. Our findings reveal that these moth species tend to initiate their spring flights within days of one another, and that there is a high degree of synchrony (overlap) among the flights of these species. However, the peak flights among these species tend to be different from one another. Mid-flight treatment timings, therefore, may need to be tailored to each species, depending on the type of control tactic and stage of the targeted species. Using DD accumulations for key biological events for each species, pest control programs can be tailored to suit the particular needs of any given marsh.

## Figures and Tables

**Table 1:** Degree-day accumulation (mean ± 95% CI) associated with the peak of each species’ respective flight. Degree-day values are derived from the *x*-maxima of the models listed in Tables S1–S3. The corresponding graphical representations of these models are presented in Figures S1–S6.

Moth Species	South	Central	East	All Regions
*A. vaccinii*	1714.9	1776.3	1801.7	1760.9 ± 44.0
*S. sulfureana*	1571.7	1622.9	1638.3	1608.9 ± 59.8
*R. naevana*	1335.7	1402.3	1350.2	1357.8 ± 26.5

## Supplementary Materials

The following are available online at http://www.mdpi.com/2075-4450/8/1/26/s1, Figure S1: Modeled flight dynamics of cranberry fruitworm (*A. vaccinii*) in the growing seasons of 2003, 2005, 2007; Figure S2: Modeled flight dynamics of cranberry fruitworm (*A. vaccinii*) in the growing seasons of 2008–2011; Figure S3: Modeled flight dynamics of sparganothis fruitworm (*S. sulfureana*) in the growing seasons of 2003, 2005, 2007; Figure S4: Modeled flight dynamics of sparganothis fruitworm (*S. sulfureana*) in the growing seasons of 2008–2011; Figure S5: Modeled flight dynamics of blackheaded fireworm (*R. naevana*) in the 2003, 2005, and 2007 growing seasons; Figure S6: Modeled flight dynamics of blackheaded fireworm (*R. naevana*) in the growing seasons of 2008–2011; Figure S7: Flight dynamics of cranberry fruitworm (CFW), sparganothis fruitworm (SFW), and blackheaded fireworm (BHFW) in the Tomah area (southern region); Figure S8: Flight dynamics of cranberry fruitworm (CFW), sparganothis fruitworm (SFW), and blackheaded fireworm (BHFW) in the Hancock area (central region); Figure S9: Flight dynamics of cranberry fruitworm (CFW), sparganothis fruitworm (SFW), and blackheaded fireworm (BHFW) in the Wisconsin Rapids area (eastern region); Table S1: Cranberry fruitworm (*A. vaccinii*) peak flight, by region and year (model parameter values); Table S2: Sparganothis fruitworm (*S. sulfureana*) peak flight, by region and year (model parameter values); Table S3: Blackheaded fireworm (*R. naevana*) peak flight, by region and year (model parameter values).
