# Supplementary material for: Flight Synchrony among the Major Moth Pests of Cranberries in the Upper Midwest, USA
Source: Insects. 2017 Feb 26;8(1):26. doi: 10.3390/insects8010026 (PMC5371954; doi:10.3390/insects8010026)
Supplement: Supplementary file 1 [file insects-08-00026-s001.pdf]

Supplementary Materials: Flight Synchrony among the Major Moth Pests of Cranberries in the Upper Midwest, USA

Shawn A. Steffan, Merritt E. Singleton, Jayne Sojka, Elissa M. Chasen, Annie E. Deutsch, Juan E. Zalapa and Christelle Guédot

Table S1. Cranberry fruitworm (*A. vaccinii*) peak flight, by region and year (model parameter values).

Region	Year	Model	y'	x (Maxima)
Central	2003	$y = ax^2 + bx + c$	$y' = 2(-0.000053528)x + 0.1809$	1690.654
Central	2005	$y = ax^2 + bx + c$	$y' = 2(-0.000034738)x + 0.1347$	1938.799
Central	2007	$y = ax^2 + bx + c$	$y' = 2(-0.000077874)x + 0.2916$	1872.255
Central	2008	$y = ax^2 + bx + c$	$y' = 2(-0.000065523)x + 0.2148$	1639.119
Central	2009	$y = ax^2 + bx + c$	$y' = 2(-0.000088068)x + 0.307$	1742.97
Central	2010	$y = ax^2 + bx + c$	$y' = 2(-0.0000221221)x + 0.0752$	1771.913
Central	2011	$y = ax^2 + bx + c$	$y' = 2(-0.000035286)x + 0.1255$	1778.376
East	2003	$y = ax^2 + bx + c$	$y' = 2(-0.000084052)x + 0.3047$	1812.568
East	2005	$y = ax^2 + bx + c$	$y' = 2(-0.000052128)x + 0.1979$	1898.212
East	2007	$y = ax^2 + bx + c$	$y' = 2(-0.000038860)x + 0.1426$	1834.792
East	2008	$y = ax^2 + bx + c$	$y' = 2(-0.000049840)x + 0.1605$	1610.152
East	2009	$y = ax^2 + bx + c$	$y' = 2(-0.0001)x + 0.4481$	2240.5
East	2010	$y = ax^2 + bx + c$	$y' = 2(-0.000064870)x + 0.2402$	1851.395
East	2011	$y = ax^2 + bx + c$	$y' = 2(-0.000073308)x + 0.2644$	1803.35
South	2003	$y = ax^2 + bx + c$	$y' = 2(-0.000074187)x + 0.2832$	1908.69
South	2005	$y = ax^2 + bx + c$	$y' = 2(-0.0000308)x + 0.1167$	1894.481
South	2007	$y = ax^2 + bx + c$	$y' = 2(-0.000051903)x + 0.1813$	1746.527
South	2008	$y = ax^2 + bx + c$	$y' = 2(-0.00008203)x + 0.2568$	1565.281
South	2009	$y = ax^2 + bx + c$	$y' = 2(-0.0001)x + 0.3321$	1660.5
South	2010	$y = ax^2 + bx + c$	$y' = 2(-0.000025695)x + 0.0818$	1591.749
South	2011	$y = ax^2 + bx + c$	$y' = 2(0.001)x + 0.0000012216$	1637.197

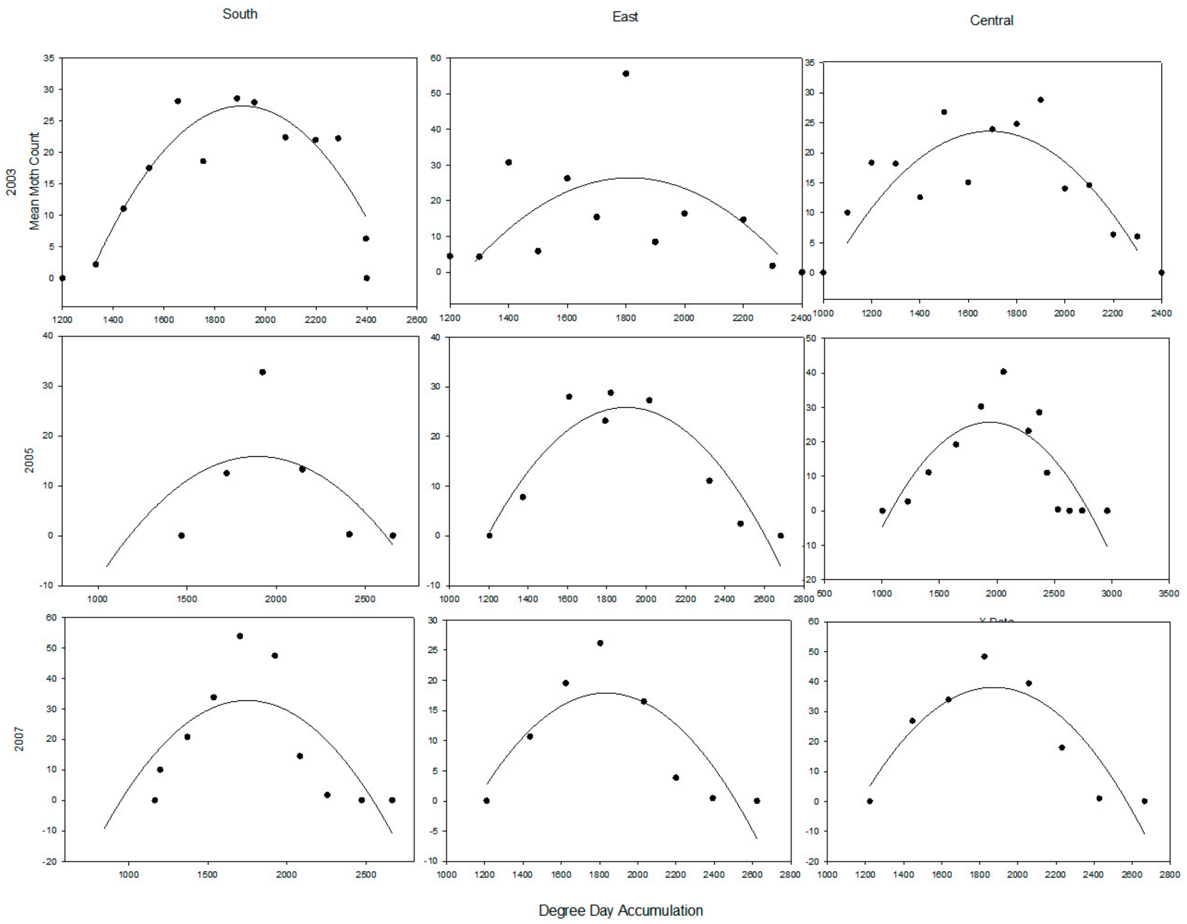

Figure S1. Modeled flight dynamics of cranberry fruitworm (*A. vaccinii*) in the growing seasons of 2003, 2005, 2007.

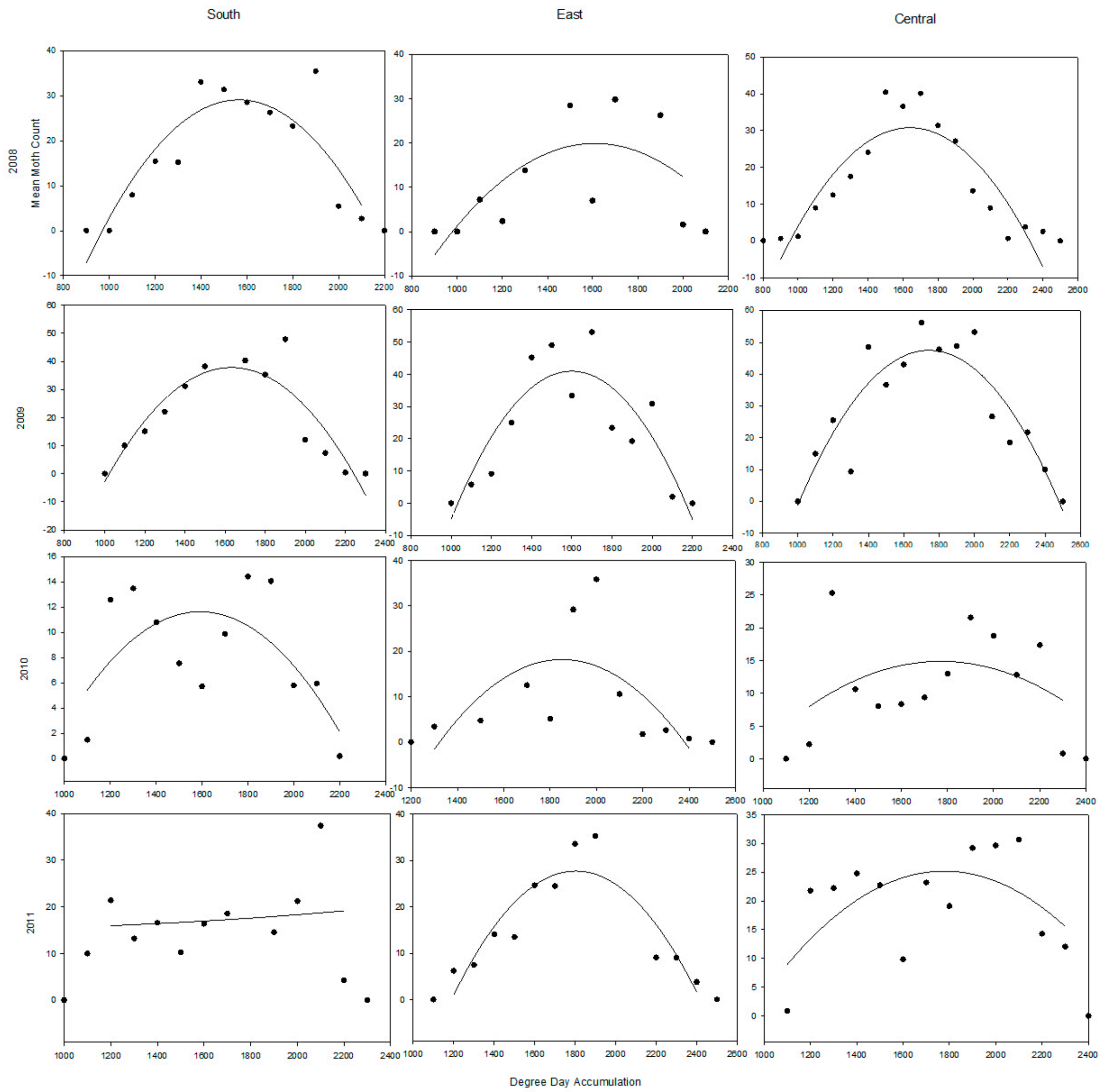

Figure S2. Modeled flight dynamics of cranberry fruitworm (*A. vaccinii*) in the growing seasons of 2008–2011.

Table S2. Sparganothis fruitworm (*S. sulfureana*) peak flight, by region and year (model parameter values).

Region	Year	Model	y'	X(maxima)
South	2003	$y = ax^2 + bx + c$	$y' = 2(-0.000099033)x + 0.3604$	1817.76
South	2005	$y = ax^2 + bx + c$	$y' = 2(-0.00009998)x + 0.3605$	1802.86
South	2007	$y = ax^2 + bx + c$	$y' = 2(-0.0001089)x + 0.3266$	1499.76
South	2008	$y = ax^2 + bx + c$	$y' = 2(-0.000132)x + 0.37833$	1433.07
South	2009	$y = ax^2 + bx + c$	$y' = 2(-0.000097173)x + 0.2852$	1467.85
South	2010	$y = ax^2 + bx + c$	$y' = 2(-0.00009117)x + 0.2618$	1435.764
South	2011	$y = ax^2 + bx + c$	$y' = 2(-0.000087334)x + 0.2698$	1544.64
East	2003	$y = ax^2 + bx + c$	$y' = 2(-.0001)x + 0.3498$	1749
East	2005	$y = ax^2 + bx + c$	$y' = 2(-0.0001)x + 0.3865$	1932.5
East	2007	$y = ax^2 + bx + c$	$y' = 2(-0.000081284)x + 0.2627$	1615.939
East	2008	$y = ax^2 + bx + c$	$y' = 2(-0.000045593(x + 0.1428$	1552.41
East	2009	$y = ax^2 + bx + c$	$y' = 2(-0.000083105)x + 0.2467$	1484.267
East	2010	$y = ax^2 + bx + c$	$y' = 2(-0.000094674)x + 0.3080$	1626.63

East	2011	$y = ax^2 + bx + c$	$y' = 2(-0.000075071)x + 0.2705$	1801.628
Central	2003	$y = ax^2 + bx + c$	$y' = 2(-0.0001)x + 0.3380$	1690
Central	2005	$y = ax^2 + bx + c$	$y' = 2(-0.000116)x + 0.4182$	1802.52
Central	2007	$y = ax^2 + bx + c$	$y' = 2(-0.0001)x + 0.3289$	1644.5
Central	2008	$y = ax^2 + bx + c$	$y' = 2(-0.000069171)x + 0.1979$	1430.513
Central	2009	$y = ax^2 + bx + c$	$y' = 2(-0.000097031)x + 0.2972$	1531.469
Central	2010	$y = ax^2 + bx + c$	$y' = 2(-0.000067664)x + 0.2217$	1638.24
Central	2011	$y = ax^2 + bx + c$	$y' = 2(-0.0001)x + 0.3731$	1865.5

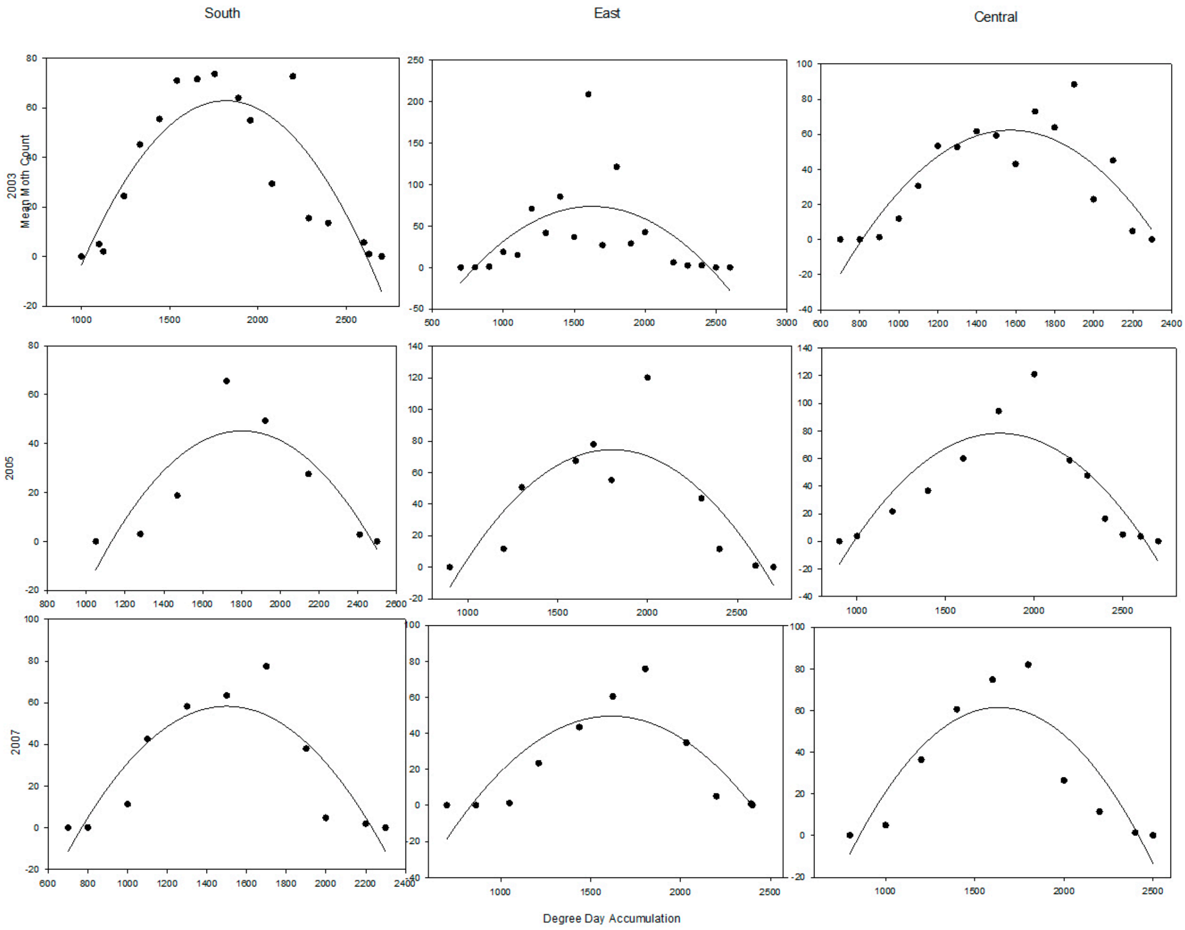

Figure S3. Modeled flight dynamics of sparganothis fruitworm (*S. sulfureana*) in the growing seasons of 2003, 2005, 2007.

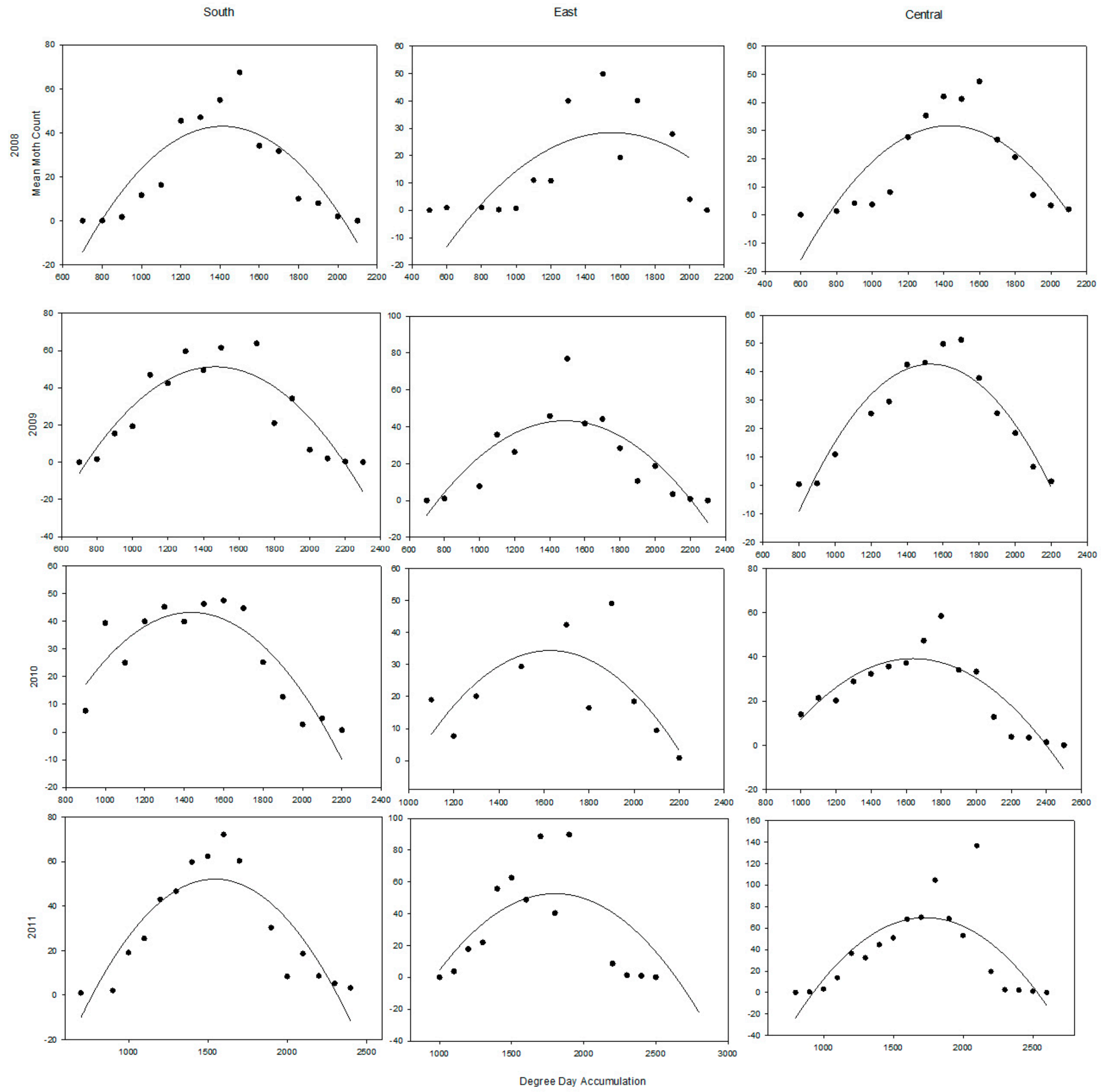

Figure S4. Modeled flight dynamics of sparganothis fruitworm (*S. sulfureana*) in the growing seasons of 2008–2011.

Table S3. Blackheaded fireworm (*R. naevana*) peak flight, by region and year (model parameter values).

Region	Year	Model	y'	x (Maxima)
Central	2003	$y = ax^2 + bx + c$	$y' = 2(-0.000029021)x + 0.0811$	1397.2641
Central	2005	$y = ax^2 + bx + c$	$y' = 2(-0.0000069093)x + 0.0210$	1519.69
Central	2007	$y = ax^2 + bx + c$	$y' = 2(-0.0000098178)x + 0.0277$	1410.703
Central	2008	$y = ax^2 + bx + c$	$y' = 2(-0.000026491)x + 0.0655$	1236.2689
Central	2009	$y = ax^2 + bx + c$	$y' = 2(-0.000068155)x + 0.1777$	1303.646
Central	2010	$y = ax^2 + bx + c$	$y' = 2(-0.000024650)x + 0.0729$	1478.7018
Central	2011	$y = ax^2 + bx + c$	$y' = 2(-0.000023510)x + 0.0691$	1469.5874
East	2003	$y = ax^2 + bx + c$	$y' = 2(-0.000011532)x + 0.0322$	1396.115
East	2005	$y = ax^2 + bx + c$	$y' = 2(-0.0000025891)x + 0.0069$	1332.509
East	2007	$y = ax^2 + bx + c$	$y' = 2(-0.0000084126)x + 0.0227$	1349.263
East	2008	$y = ax^2 + bx + c$	$y' = 2(-0.000009899)x + 0.0271$	1368.825
East	2009	$y = ax^2 + bx + c$	$y' = 2(-0.000012267)x + 0.032$	1304.3124
East	2010	$y = ax^2 + bx + c$	$y' = 2(-0.0000070582)x + 0.0236$	1666.196

East	2011	$y = ax^2 + bx + c$	$y' = 2(-0.000015679)x + 0.0547$	1744.3715
South	2003	$y = ax^2 + bx + c$	$y' = 2(-0.00039717)x + 0.1218$	1533.348
South	2005	$y = ax^2 + bx + c$	$y' = 2(-0.000014195)x + 0.0446$	1570.9757
South	2007	$y = ax^2 + bx + c$	$y' = 2(-0.000024591)x + 0.0653$	1327.7215
South	2008	$y = ax^2 + bx + c$	$y' = 2(-0.00001975)x + 0.0499$	1263.29
South	2009	$y = ax^2 + bx + c$	$y' = 2(-0.000031389)x + 0.0814$	1296.8392
South	2010	$y = ax^2 + bx + c$	$y' = 2(-0.0000050784)x + 0.0146$	1437.46
South	2011	$y = ax^2 + bx + c$	$y' = 2(-0.000026867)x + 0.0727$	1352.96

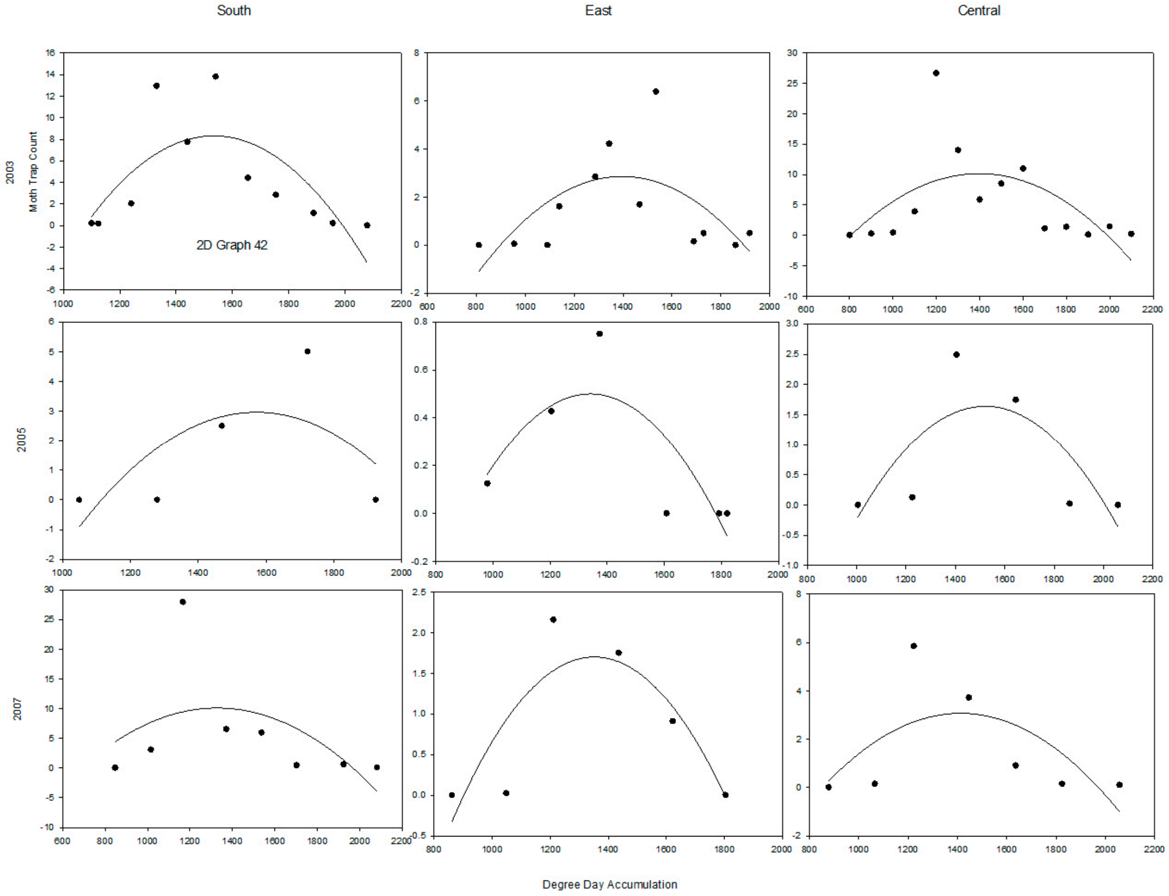

Figure S5. Modeled flight dynamics of blackheaded fireworm (*R. naevana*) in the 2003, 2005, and 2007 growing seasons.

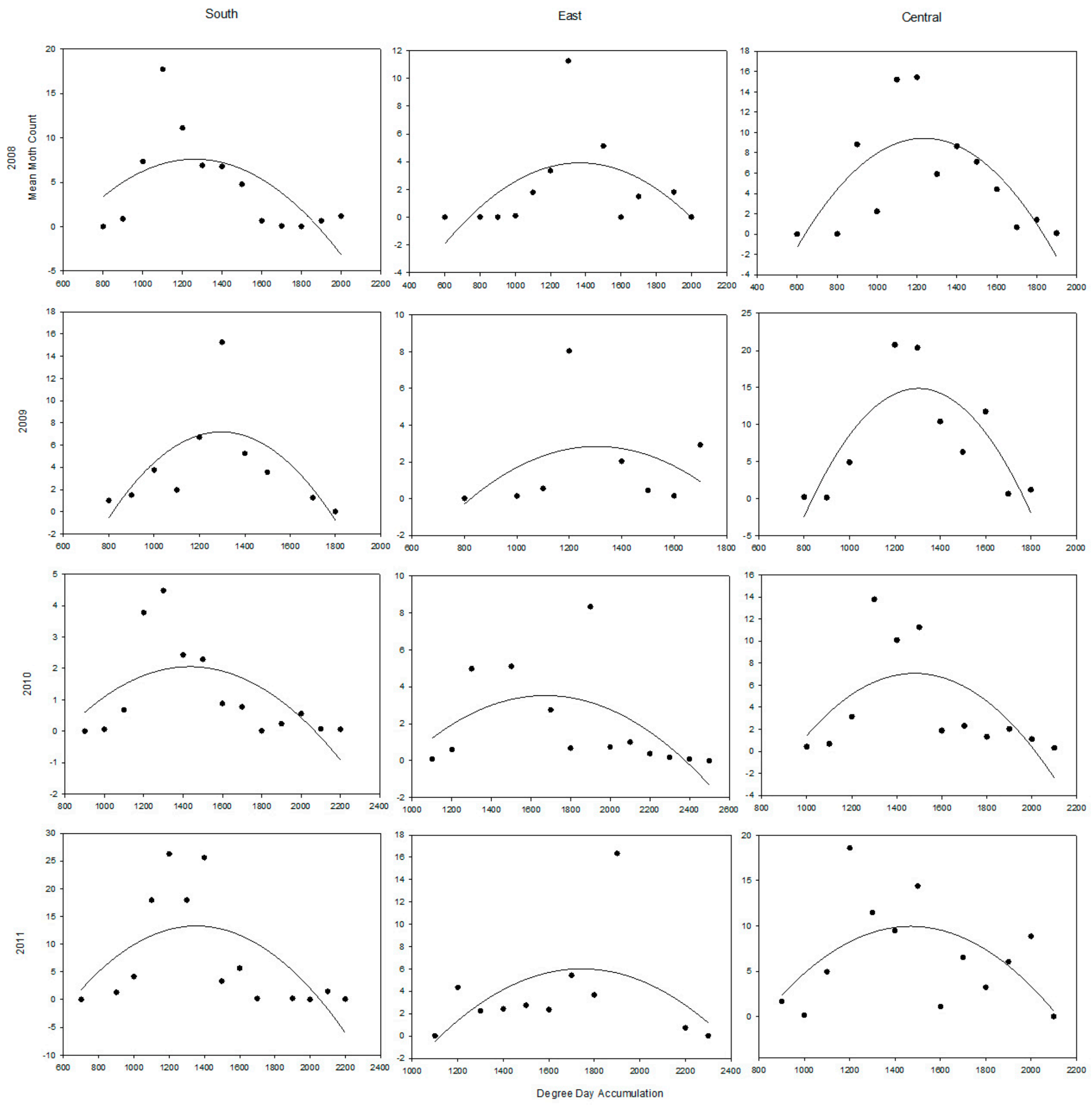

Figure S6. Modeled flight dynamics of blackheaded fireworm (*R. naevana*) in the growing seasons of 2008–2011.

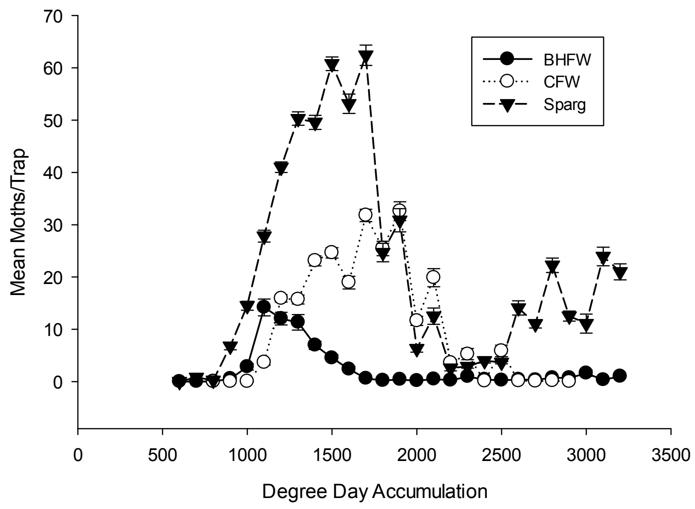

Figure S7. Flight dynamics of cranberry fruitworm (CFW), sparganothis fruitworm (SFW), and blackheaded fireworm (BHFW) in the Tomah area (southern region).

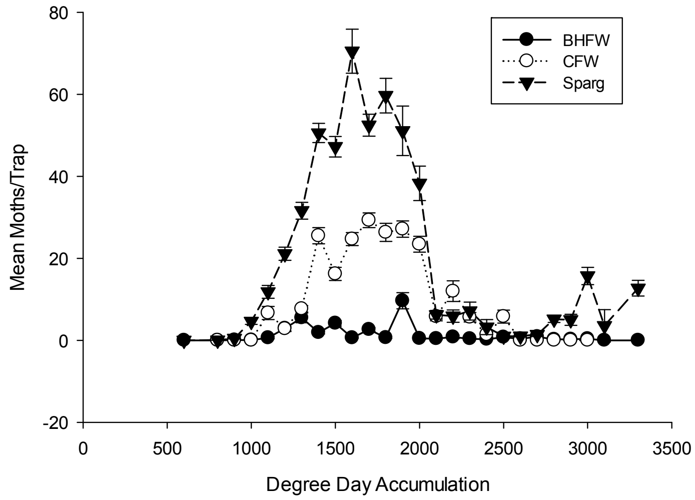

Figure S8. Flight dynamics of cranberry fruitworm (CFW), sparganothis fruitworm (SFW), and blackheaded fireworm (BHFW) in the Hancock area (central region).

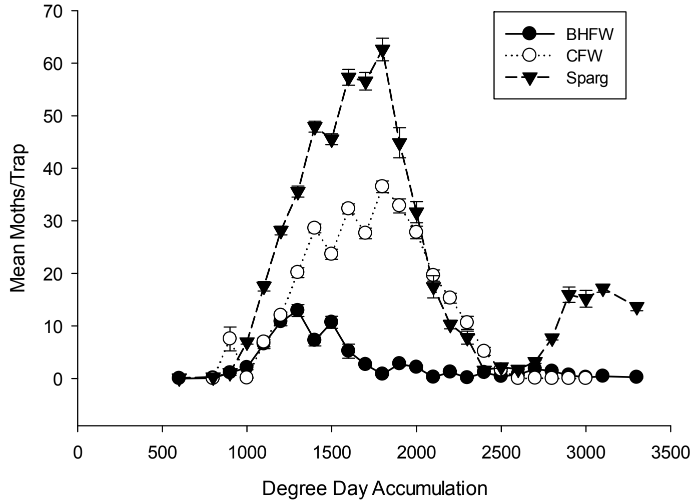

Figure S9. Flight dynamics of cranberry fruitworm (CFW), sparganothis fruitworm (SFW), and blackheaded fireworm (BHFW) in the Wisconsin Rapids area (eastern region).
